# Supplementary material for: The diagnostic utility of glycosaminoglycans (GAGs) in the early detection of cancer: a systematic review
Source: PeerJ. 2024 Nov 21;12:e18486. doi: 10.7717/peerj.18486 (PMC11586047; doi:10.7717/peerj.18486)
Supplement: Supplemental Information 1 [file peerj-12-18486-s001.docx]

*Table S1 – CASP Table (Supplementary)*

| **Case Control Studies** | | | | | | | | | | | | | |
| --- | --- | --- | --- | --- | --- | --- | --- | --- | --- | --- | --- | --- | --- |
| **Author/ Year** | Did the study address a clearly focused issue? | Did the authors use an appropriate method to answer their question? | Were the cases recruited in an acceptable way? | Were the controls selected in an acceptable way? | Was the exposure accurately measured to minimize bias? | Have the authors taken account of the potential confounding factors in the design and/or in their analysis? | How large was the treatment effect? | How precise was the estimate of the treatment effect? | Do you believe the results? | Can the results be applied to the local population? | Do the results of this study fit with other available evidence? | Aside from the experimental intervention, were the groups treated equally? | Grade |
| **Biskup et al (2021) (9)** | Yes  1 | Yes  1 | Can't tell  0.5 | Can't tell  0.5 | Yes  1 | Yes  1 | Can't tell  0.5 | Yes  1 | Yes  1 | Can't tell  0.5 | Can't tell  0.5 | Can't tell  0.5 | 9/12 |
| **da Silva et al (2018)**  **(10)** | Yes  1 | Yes  1 | Can't tell  0.5 | No  0 | Yes  1 | No  0 | Can't tell  0.5 | Can't tell  0.5 | Yes  1 | No  0 | Yes  1 | No  0 | 6.5/12 |
| **Gatto et al (2016)**  **(7)** | Yes  1 | Can't tell  0.5 | Yes  1 | Yes  1 | Yes  1 | Can’t tell  0.5 | Can't tell  0.5 | Yes  1 | Yes  1 | Yes  1 | Can't tell  0.5 | No  0 | 9/12 |
| **Aghcheli et al**  **(2012)**  **(17)** | Yes  1 | Yes  1 | Can’t tell  0.5 | Can’t Tell  0.5 | Yes  1 | No  0 | Can’t Tell 0.5 | Yes  1 | Yes  1 | Yes  1 | Can’t tell  0.5 | Can 'tell  0.5 | 8.5/12 |
| **Xing et al**  **(2008)**  (14) | Yes1 | Yes  1 | Can't tell  0.5 | Can't tell  0.5 | Yes  1 | Yes  1 | Can't tell  0.5 | Yes  1 | Yes  1 | Can't tell  0.5 | Can't tell  0.5 | Can't tell  0.5 | 9/12 |
| **Gatto et al**  **(2018)**  (8) | Yes  1 | Yes  1 | Can’t tell  0.5 | Can’t Tell  0.5 | Yes  1 | No  0 | Can’t Tell 0.5 | Yes  1 | Yes  1 | Yes  1 | Can’t tell  0.5 | Can 'tell  0.5 | 8.5/12 |
| **Cross sectional Studies** | | | | | | | | | | | | | |
|  | Did the study address a clearly focused issue? | Was the cohort recruited in an acceptable way? | Was the exposure accurately measured to minimize bias? | Was the outcome measured correctly to minimize bias? | Have the authors identified all the important confounding factors? | Have they taken account of confounding factors in the design and/or analysis? | Was the follow up of subjects complete enough? | Was the follow up of subjects long enough? | Do you believe in the results? | Can the results be applied to the local population? | Do the results of this study fit with other evidence? | What are the implications of this study for practice? | Grade |
| **El-Mezayen et al (2012)(12)** | Yes  1 | Yes  1 | Yes  1 | Yes  1 | Yes  1 | Yes  1 | Can't tell  0.5 | No  0 | Yes  1 | Yes  1 | Can't tell  0.5 | Yes  1 | 10/12 |
| **Gatto et al (2022)**  **(11)** | Yes  1 | Yes  1 | Yes  1 | Yes  1 | Yes  1 | Can’t tell  0.5 | Yes  1 | Can’t tell  0.5 | Yes  1 | Can’t tell  0.5 | Yes  1 | Yes  1 | 10.5/12 |
| **Zhang et al (2019)**  **(18)** | Yes  1 | Yes  1 | Can't tell  0.5 | No  0 | Yes  1 | No  0 | Can't tell  0.5 | Yes  1 | Yes  1 | Can't tell  0.5 | Can't tell  0.5 | Can't tell  0.5 | 7.5/12 |
| **Creaney et al**  **(2013)**  **(19)** | Yes  1 | Yes  1 | Yes  1 | Yes  1 | Yes  1 | Yes  1 | Can't tell  0.5 | No  0 | Yes  1 | Can't tell  0.5 | Can't tell  0.5 | Yes  1 | 9.5/12 |
| **Rangel et al**  **(2015)**  **(20)** | Yes  1 | Yes  1 | No  0 | No  0 | Yes  1 | Yes  1 | Can't tell  0.5 | No  0 | Yes  1 | Yes  1 | Can't tell  0.5 | Yes  1 | 8/12 |

| **Case Control Studies** | | | | | | | | | | | | | |
| --- | --- | --- | --- | --- | --- | --- | --- | --- | --- | --- | --- | --- | --- |
| **Author/ Year** | Did the study address a clearly focused issue? | Did the authors use an appropriate method to answer their question? | Were the cases recruited in an acceptable way? | Were the controls selected in an acceptable way? | Was the exposure accurately measured to minimize bias? | Have the authors taken account of the potential confounding factors in the design and/or in their analysis? | How large was the treatment effect? | How precise was the estimate of the treatment effect? | Do you believe the results? | Can the results be applied to the local population? | Do the results of this study fit with other available evidence? | Aside from the experimental intervention, were the groups treated equally? | Grade |
| **Biskup et al (2021) (8)** | Yes  1 | Yes  1 | Can't tell  0.5 | Can't tell  0.5 | Yes  1 | Yes  1 | Can't tell  0.5 | Yes  1 | Yes  1 | Can't tell  0.5 | Can't tell  0.5 | Can't tell  0.5 | 9/12 |
| **da Silva et al (2018)**  **(9)** | Yes  1 | Yes  1 | Can't tell  0.5 | No  0 | Yes  1 | No  0 | Can't tell  0.5 | Can't tell  0.5 | Yes  1 | No  0 | Yes  1 | No  0 | 6.5/12 |
| **Gatto et al (2016)**  **(6)** | Yes  1 | Can't tell  0.5 | Yes  1 | Yes  1 | Yes  1 | Can’t tell  0.5 | Can't tell  0.5 | Yes  1 | Yes  1 | Yes  1 | Can't tell  0.5 | No  0 | 9/12 |
| **Yang et al (2022)**  **(7)** | Yes  1 | Yes  1 | No  0 | No  0 | Yes  1 | No  0 | Can't tell  0.5 | Can't tell  0.5 | Yes  1 | Can't tell  0.5 | Yes  1 | Can't tell  0.5 | 7/9 |
| **Cross sectional Study** | | | | | | | | | | | | | |
|  | Did the study address a clearly focused issue? | Was the cohort recruited in an acceptable way? | Was the exposure accurately measured to minimize bias? | Was the outcome measured correctly to minimize bias? | Have the authors identified all the important confounding factors? | Have they taken account of confounding factors in the design and/or analysis? | Was the follow up of subjects complete enough? | Was the follow up of subjects long enough? | Do you believe in the results? | Can the results be applied to the local population? | Do the results of this study fit with other evidence? | What are the implications of this study for practice? |  |
| **Svensson et al (2011)**  **(13)** | Yes.  1 | Yes  1 | No  0 | Yes  1 | No  0 | No  0 | Yes  1 | Yes  1 | Can’t tell  0.5 | Yes  1 | Yes  1 | Yes  1 | 8.5/12 |
| **Kramer et al (2010)**  **(11)** | Yes  1 | Yes  1 | Yes  1 | Yes  1 | Yes  1 | Yes  1 | Can't tell  0.5 | Yes  1 | Yes  1 | Yes  1 | Can't tell  0.5 | Yes  1 | 11/12 |
| **El-Mezayen et al (2012)**  **(10)** | Yes  1 | Yes  1 | Yes  1 | Yes  1 | Yes  1 | Yes  1 | Can't tell  0.5 | No  0 | Yes  1 | Yes  1 | Can't tell  0.5 | Yes  1 | 10/12 |
| **Gatto et al (2022)**  **(12)** | Yes  1 | Yes  1 | Yes  1 | Yes  1 | Yes  1 | Can’t tell  0.5 | Yes  1 | Can’t tell  0.5 | Yes  1 | Can’t tell  0.5 | Yes  1 | Yes  1 | 10.5/12 |
| **Peng et al (2016)**  **(14)** | Yes  1 | Yes  1 | Yes  1 | Can’t tell  0.5 | Yes  1 | No  0 | Can't tell  0.5 | Can't tell  0.5 | Yes  1 | Yes  1 | Yes  1 | Can't tell  0.5 | 9/12 |
| **Diagnostic Studies** | | | | | | | | | | | | | |
|  | Was there a clear question for the study to address? | Was there a comparison with an appropriate reference standard? | Did all patients get the diagnostic test and reference standard? | Could the results of the test have been influenced by the results of the reference standard? | Is the disease status of the tested population clearly described? | Were the methods for performing the test described in sufficient detail? | Can the results be applied to your patients/the population of interest? | Can the test be applied to your patient or population of interest? | Were all outcomes important to the individual or population considered? |  |  |  |  |
| **Purushothaman et al (2017) (15)** | Can’t tell  0.5 | No  0 | No  0 | No  0 | No  0 | Yes  1 | Can’t tell  0.5 | Yes  1 | Yes  1 |  |  |  | 4/9 |
| **Wang et al (2013)**  **(16)** | Yes  1 | Yes  1 | No  0 | Yes  1 | Can't tell  0.5 | Yes  1 | Yes  1 | Yes  1 | Yes  1 |  |  |  | 7.5/9 |
